# Connecting Emotional Intelligence with Academic Performance and Family Dynamics among Fresh Undergraduates in a Health Sciences University: Preliminary Findings

**DOI:** 10.4314/ejhs.v35i1.7

**Published:** 2025-01

**Authors:** Joshua Falade, Ezekiel Taiwo Adebayo, Benjamin Adekunle Eegunranti, Adesegun Olayiwola Fatusi

**Affiliations:** 1 Department of Mental Health, Faculty of Clinical Sciences, University of Medical Sciences, Ondo, Ondo State, Nigeria; 2 Department of Oral and Maxillofacial Surgery, Faculty of Dental Sciences, College of Health Science, University of Medical Sciences, Ondo, Ondo State, Nigeria; 3 Department of Psychiatry, Faculty of Clinical Science, Ladoke Akintola University of Technology, Ogbomoso, Oyo State, Nigeria; 4 Department of Community Medicine, College of Health Science, University of Medical Science Ondo

**Keywords:** Emotional intelligence, Family dynamics, Psychiatric morbidity, Academic performance, Fresh undergraduates, Health sciences

## Abstract

**Background:**

Emotional intelligence (EI) is vital in various life domains, particularly for new undergraduates in health sciences. The study assessed the prevalence and associated factors of above-average EI among fresh undergraduates in a health sciences university.

**Methods:**

A cross-sectional descriptive study was conducted at UNIMED Ondo, assessing socio-demographic characteristics, family dynamics, psychiatric morbidity, and EI through questionnaires. Data were analyzed with SPSS 21 from November 2023 to January 2024.

**Results:**

The prevalence of above-average EI was 92.9%. Respondents with married parents had significantly higher odds of above-average EI compared to those with non-married parents (odds ratio: 13.466, p < 0.001). Respondents who self-identified as academically “good” had 0.059 times lower odds of above-average EI compared to those who identified as “very good” academically (p < 0.001). The odds of above-average EI increased by one for each scale increase in monthly allowance (p = 0.034). Conversely, the odds decreased by 0.889 for each scale increase in Post United Matriculation Examination score (p < 0.001). The odds of above-average EI also decreased by 0.481 for each scale increase in the number of children the mother had (OR = 0.481, p < 0.001).

**Conclusions:**

Fresh health sciences undergraduates demonstrated high EI, which positively impacted academic performance . Strengthening family systems is critical for enhancing EI.

## Introduction

The concept of intelligence has existed for as long as human history and is closely tied to cognitive skills (1). In addition to cognitive intelligence, other types of intelligence, such as emotional, social, and financial intelligence, have been identified as indicators of overall adjustment (2). Emotional intelligence (EI), as defined by Salovey and Mayer, refers to the ability to monitor one's own and others' emotions, discriminate among them, and use this information to guide thinking and action(3). Researchers have proposed a four-branch ability theory of EI, which categorizes EI into perceiving emotion, using emotion to facilitate thought, understanding emotions, and managing emotions. These branches reflect the progression of emotional intelligence skills from perception to management, ultimately shaping a person's personality. EI has been shown to be a critical factor in academic success and overall achievement in various educational settings (4).

A study found that emotional intelligence positively influenced academic performance among medical undergraduates, with higher EI linked to lower levels of perceived stress and better academic outcomes(5). Other studies, including one in Sri Lanka, also found a significant correlation between EI and academic performance among medical students. (6) Similarly, research in Pakistan indicated that EI positively impacted academic success among undergraduates (7). These findings emphasize the importance of EI in academic settings and its role in academic achievement.

Emotional intelligence has also been shown to correlate with mental well-being, with high EI serving as a protective factor against psychological risks, such as burnout and psychosomatic complaints (8). For health sciences students, EI has been linked to better mental health outcomes. Family structure also plays a significant role in emotional intelligence, with a stable and supportive family environment fostering the development of healthy emotional skills (9). Authoritative parenting, in particular, has been positively correlated with higher EI in children, while authoritarian and permissive parenting styles are linked to lower EI (10).

In Nigeria, low EI among undergraduates has been shown to negatively impact academic performance and personal development, as students with low EI experience higher levels of anxiety and poor academic outcomes. EI, which involves recognizing and managing emotions, is crucial for effective communication and interpersonal relationships (11). Low EI among students also affects leadership skills, conflict resolution, and other life skills. As studies show a strong relationship between EI and academic success, integrating EI development into educational curricula could help nurture well-rounded graduates capable of excelling both academically and professionally (12).

Despite its importance, the educational system has traditionally focused more on developing IQ rather than EI. Standardized IQ tests have long been used as the primary measure of academic success, emphasizing cognitive abilities over emotional development (13). This focus on IQ often overlooks the significance of emotional skills, such as empathy and resilience, which are vital for personal and professional success (14).

Furthermore, societal expectations have reinforced this emphasis on IQ. Parents, educators, and administrators often prioritize academic achievement based on test scores and grades, diminishing the perceived value of EI (15). This systemic focus on IQ can hinder the development of essential soft skills that are crucial for navigating complex personal and professional challenges.

While there is extensive literature on the relationship between EI, academic performance, family dynamics, and psychiatric morbidity, there is a gap in understanding the prevalence and predictors of EI among health sciences undergraduates. This study aims to fill that gap, providing valuable insights for improving training and reducing psychiatric morbidity in healthcare professionals.

This study aimed to examine the relationship between emotional intelligence (EI), psychiatric morbidity, and academic performance among fresh undergraduates at a Nigerian health sciences university. Specifically, it evaluated the prevalence of EI and its associations with academic achievement, family dynamics, and psychiatric morbidity.

## Materials and Methods

The study was conducted at the University of Medical Sciences Ondo (UNIMED), involving all fresh undergraduates enrolled in the 2023/2024 academic session. This cross-sectional study used a self-administered questionnaire to collect data.

The questionnaire covered socio-demographic details, family dynamics, and the monthly allowance received by students. It also assessed respondents' self-evaluated academic status. EI was measured using the Wong and Law Emotional Intelligence Scale, while psychiatric morbidity was assessed with the General Health Questionnaire (GHQ).

The entrance to the university was based on an aggregate score from the university's Post UTME and the Joint Admissions and Matriculation Board (JAMB) examinations. The aggregate score was calculated by dividing the UTME score by eight and the Post UTME score by two, with the maximum possible Post UTME score being 100%.

This study represents preliminary data from a five-year prospective study involving undergraduates at UNIMED. The inclusion criteria were limited to fresh undergraduates who provided informed consent.

**Sampling method and procedure**: A census approach was employed, using a cross-sectional descriptive design. Participants were randomly recruited from those undergoing routine medical screening. Consent was obtained from students who met the inclusion criteria. Participants completed self-administered questionnaires, which were then collected by the researchers and research assistants. Any incomplete questionnaires were addressed with the help of trained research assistants. Post-UTME scores were obtained from university records. The study was conducted from November 2023 to January 2024.

**Emotional intelligence questionnaire**: EI was measured using the Wong and Law Emotional Intelligence Scale (WLEIS), a widely used tool based on the ability model of EI. The scale has four dimensions: self-emotional appraisal (SEA), others' emotional appraisal (OEA), regulation of emotion (ROE), and use of emotion (UOE) (3). It has been validated in Nigeria (16) with a high reliability (Cronbach's alpha = 0.92) from a pilot study among selected 50 secondary school teacher. Respondents rated their agreement with 16 statements on a 7-point scale, from “strongly disagree” to “strongly agree.” (17) A total EI score was computed, and respondents scoring 50% or more (48 points) were classified as having above-average EI.

**The General Health Questionnaire (GHQ)**: The GHQ-12, designed by David Goldberg, is a self-administered screening tool used to detect non-psychotic psychiatric disorders (18). It uses a binary scoring system (0-0-1-1), with a score of 3 or above indicating psychiatric morbidity (19). In previous studies, the GHQ-12 has shown good reliability (alpha coefficient = 0.82 (20).

**Data analysis**: Data were analyzed using SPSS version 21. Descriptive statistics, such as frequencies, means, and standard deviations, were used to summarize the socio-demographic details. Chi-square tests were performed to explore the associations between socio-demographic factors and EI, while logistic regression identified independent predictors of EI. A 95% confidence interval was used, with statistical significance set at p ≤ 0.05.

**Ethical considerations**: Ethical approval was granted by the Research Ethics Committee of the University of Medical Sciences Teaching Hospital, Ondo, Nigeria (UNIMEDTHC/EC/24/013). Participation was voluntary, and informed consent was obtained from all participants.

## Results

A total of 560 out of 580 respondents completed and returned the questionnaire, yielding a response rate of 96.6%. The majority of respondents (63.6%) were female, resulting in a female-to-male ratio of 1.74:1. Of the respondents, 72.5% considered themselves academically “very good,” and 94.1% were satisfied with their monthly allowance. Most respondents came from monogamous family structures, with parents typically in marital unions. The mean age of respondents was 16.9 years, and the average monthly allowance was N22,723.00. The average number of children in respondents' families was 3.8 on the father's side and 3.5 on the mother's side. The average post-UTME score was 54.2. (Table 1)

**Table 1:** Socio-demographic and family dynamic of the respondents

Variables	Frequency	Percentage
**Gender**		
Male	204	36.4
Female	356	63.6
**Academic performance**		
Good	154	27.5
Very Good	407	72.5
**Are you satisfied with your Monthly allowance**		
Yes	527	94.1
No	33	5.9
**What is your position among your father's children**		
First child	236	42.1
Others	324	57.9
**What is your position among your mother's children**		
First child	249	44.5
Others	311	55.5
**Marital Status of your parent**		
Married	487	87.0
Non married	73	13.0
**Family Type**		
Monogamy	528	94.3
Polygamy	32	5.7
**Father occupation category**		
Civil servant	317	56.6
Non-servant	232	41.4
Non-employed	11	2.0
**Mother's Occupation Category**		
Civil servant	308	55.0
Non civil servant	247	44.1
Non employed	5	0.9
**Mother's level of education**		
Graduate	464	82.9
Undergraduate	96	17.1
**Father's level of education**		
Graduate	437	78.0
Undergraduate	123	22.0
**Age (years±SD)**	16.9±1.7	
**Age range (years)**	14-26	
**Average monthly allowance (Naira)**	22723±10465	
**Average number of children of the father**	3.8±1.4	
**Average number of children of the mother**	3.5 ±1.1	
**Mean Father's Age**	52.1±6.6	
**Mean Mother's Age**	48.0 ±5.0	
**Mean Post UTME score**	54.2 ±12.0	

**Figure 1:**
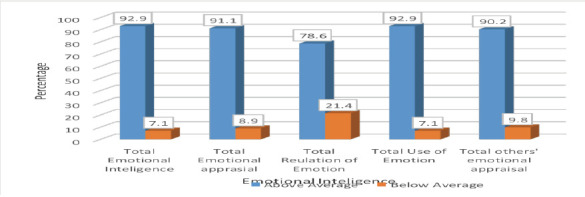
Extent of EI among the respondents

Among the respondents, 92.8% scored above-average EI, though the lowest percentages were observed for “regulation of emotion” (78.6%) and “others' emotional appraisal” (90.2%). (Figure 1) Additionally, 5.5% of the respondents had psychiatric morbidity.

**Association between socio-demographics, family dynamics, and EI**: A significant association was found between self-perceived academic performance and EI. 95.6% of respondents who considered themselves “very good” academically exhibited above-average EI, compared to 85.7% of those who considered themselves “good” (X^2^ = 16.340, p = 0.001). Similarly, parental marital status was strongly associated with EI. Respondents with married parents showed higher rates of above-average EI (95.9%) compared to those with non-married parents (72.6%) (X^2^ = 51.920, p = 0.001). (Table 2)

**Table 2:** Association between the socio-demographic, family dynamics and EI using Chi- square

Variable	Below average	above average	*P value*
Gender			
Male	18(8.8%)	186(91.2%)	0.242
Female	22(6.2%)	334(93.5%)	
Academic Performance			
Good	22(14.3%)	132(85.7%)	0.001
Very Good	18(4.4%)	388(95.6%)	
Are you satisfied with your monthly allowance?			
Yes	40(7.6%)	487(92.4%)	0.101
No	0(0.0%)	33(100.0%)	
What is your position among your fathers' children?			
First child	18(7.6%)	218(92.2%)	0.704
Others	22(6.8%)	302(93.2%)	
What is your position among your mothers' children?			
First child	18(7.2%)	231(92.8%)	0.944
Other	22(7.1%)	289(92.9%)	
Marital status of the parents			
Married	20(4.1%)	467(95.9%)	**0.001**
Non married	20(27.4%)	53(72.6%)	
Family type			
Monogamy	39(7.4%)	489(92.6%)	0.363
Polygamy	1(3.1%)	31(96.9%)	

Father occupation category			
Civil servant	18(5.7%)	299(94.3%)	0.151
Non civil servant	22(9.5%)	210(90.5%)	
Non employed	0(0.0%)	11(100.0%)	

Mother occupation category			
Civil servant	26(8.4%)	282(91.6%)	0.372
Non civil servant	14(5.7%)	233(94.3%)	
Non employed	0(0.0-%)	5(100.0%)	
Mother's level of education			
Graduate	37(8.0%)	427(92.0%)	0.093
Undergraduate	3(3.1%)	93(96.9%)	
Father's level of education			
Graduate	29(6.6%)	408(93.4%)	0.380
Undergraduate	11(8.9%)	112(91.1%)	

Respondents with above-average EI had lower average monthly allowances, fewer children from their mothers, and lower Post UTME scores compared to those with below-average EI. These associations were statistically significant (T = 2.220, p = 0.027; T = 2.764, p = 0.006; T = 4.817, p = 0.001, respectively). (Table 3)

**Table 3:** Association between the mean of the socio-demographic, family dynamics and EI using independent T-test

Variables	Below average	Above average	*P-value*
Age (years±SD)	16.4(±0.7)	16.9(±1.8)	0.087
Average monthly income (Naira±SD)	26250.0(±14489.2)	22451.9(±10057.5)	*0.027*
How many children has your father	3.6(±0.5)	3.8(±1.4)	0.269
How many children has your mother	3.9(±0.4)	3.5(±1.1)	*0.006*
Father's Age (years)	53.0(±7.6)	52.0(±6.5)	0.375
Mother's Age (years)	48.8(±5.1)	47.7(±5.0)	0.001
Post Jamb Score	62.9(±7.8)	53.6(±12.0)	*0.001*

**Association between psychiatric morbidity and EI**: Respondents without psychiatric morbidity had significantly higher rates of above-average EI (94.3%) compared to those with psychiatric morbidity (X^2^ = 31.209, p = 0.001).

**Socio-demographic and Family Predictors of EI**: The odds of having above-average EI were significantly higher among respondents with married parents (OR = 13.446, p < 0.001). Conversely, respondents who considered themselves “good” academically had a 0.059 times decreased odds of having above-average EI compared to those who saw themselves as “very good” (p< 0.001).

The odds of having above-average EI increased by one unit with each additional scale of monthly allowance (p = 0.034). However, the odds decreased by 0.889 for each increase in Post UTME score (p < 0.001). Furthermore, the odds of above-average EI decreased by 0.481 for each additional child from the mother (OR = 0.481, p < 0.001). (Table 4)

**Table 4:** Socio-demographic and family predictors of EI using logistic regression

number	Variables	Odd Ratio	95 % CI		Pvalue
			low	High	
1	**Marital status(parent)**				
	Non married(Ref)				
	Married(1)	13.466	4.752	38.157	**<0.001**
2	**Academic performance**				
	Very good(ref)				
	Good(1)	0.059	0.019	0.179	**<0.001**
3	**AMI**	1.000	1.000	1.000	**0.034**
4	**Post UTME**	0.889	0.842	0.938	**<0.001**
5	**Mother children**	0.481	0.319	0.725	**<0.001**

## Discussion

This study explored the relationship between Emotional Intelligence (EI), academic achievement, family dynamics, and psychiatric morbidity among fresh health sciences undergraduates. It further examined how sociodemographic and family factors relate to EI and identified the correlates of EI in a low- to middle-income economy. The findings provide valuable insights into the role of EI in students' academic and psychological well-being.

This study aimed to investigate the relationship between EI and academic achievement, family dynamics, and psychiatric morbidity among fresh health sciences undergraduates. It also explored the associations between high EI and various socio-demographic and family factors within the context of a low- to middle-income economy.

The average age of respondents was 16.9 years, consistent with prior findings from fresh undergraduates in Lagos, Nigeria (20). A higher proportion of respondents were female, which aligns with the growing trend of feminization in health sciences education.

As shown in Table 1, the majority of respondents' parents were literate, employed as civil servants, and graduates. Parental influence plays a crucial role in children's lives. Parents with higher education tend to place significant value on academic achievement, which can lead to higher student performance (21). Similar studies have reported that children of highly educated parents tend to achieve better academic outcomes, with maternal education being a particularly strong predictor of students' GPA (22). Most respondents came from monogamous family settings. Monogamy remains prevalent in society due to its association with emotional stability, commitment, and social acceptance (23). The academic rigor required for health sciences courses likely contributed to the respondents' strong academic performance.

Approximately 92.9% of respondents demonstrated above-average EI, a notably higher percentage than the 14.1% of clinical medical students in Ghana (24). While it is expected that EI improves with age and university education, the marked difference in the proportion of fresh undergraduates exhibiting high EI compared to clinical students may be due to variations in assessment tools and categorization. Similar studies, such as those conducted with postgraduate students at Kerman University of Medical Sciences in Iran, have reported similarly high EI scores using the Bradberry and Greaves EI questionnaire (25, 26). However, longitudinal studies are needed to track changes in EI throughout the course of health sciences education.

Despite the high overall EI levels, respondents exhibited lower emotional regulation and emotional appraisal scores. Emotional regulation is an essential aspect of human behavior, enabling individuals to manage and control their emotions effectively (27). Self-awareness plays a pivotal role in emotional regulation, helping individuals identify emotional triggers and manage their responses. The four dimensions of EI—self-awareness, self-regulation, social awareness, and relationship management—are interdependent, with accurate self-emotion appraisal being crucial for emotional regulation (28). Furthermore, understanding others' emotions is key to forming strong interpersonal relationships, which further aids in managing one's own emotions (29).

The study found a psychiatric morbidity prevalence of 5.5%, which is lower than the 25.5% reported in a similar study among undergraduates in health-related disciplines in Southwestern Nigeria (30). Reducing psychiatric morbidity can positively affect EI in several ways. Addressing mental health challenges can improve emotional regulation, enabling individuals to better express and manage their emotions, a core component of EI (31). Mental health improvement also increases self-awareness, allowing individuals to better understand their emotional responses (32). Reduced psychiatric morbidity fosters healthier interpersonal relationships, as individuals engage in more positive communication and empathy. Additionally, better mental health supports improved cognitive functioning, which enhances decision-making and problem-solving skills—key elements of EI (33).

The study identified several predictors of above-average EI, including parental marital status, self-rated academic performance, increased monthly allowance, low post-UTME scores, and mothers with fewer children. Emotional skills are shaped through parental guidance and role modeling. A stable marital environment fosters emotional bonds and trust, both of which are essential for developing EI (34). Parental communication and conflict resolution skills also influence emotional development. Smaller families with fewer children provide more individualized attention, which enhances emotional intelligence through closer emotional connections with the mother (35). Fewer siblings may also reduce sibling rivalry, allowing the child to focus on emotional development without added stressors.

Academic ability is often associated with self-confidence and resilience, both of which contribute to emotional intelligence. Self-perception of academic success can foster self-awareness and improve interpersonal communication (36). However, high IQ students may not always exhibit high EI, as perfectionism, pressure to excel, and a focus on rational thinking may hinder emotional development (37). This may partly explain the inverse relationship observed between EI scores and post-UTME scores in this study, which suggests a disconnect between academic self-perception and objective validation. Further investigation through longitudinal studies is needed to better understand this relationship.

Financial support, as indicated by an increased monthly allowance, has a positive impact on EI. Higher allowances reduce stress, improve quality of life, and facilitate emotional resilience (38). Increased financial support also encourages social interaction and enhances communication skills, which are essential for emotional development (39).

This study found that a significant proportion of respondents in a health sciences university exhibited above-average emotional intelligence, a trend that was higher than reported in other low- and middle-income countries. While most respondents demonstrated strong EI, concerns were raised about the use of emotion and emotional appraisal. The study suggests that age and academic training may enhance EI, though longitudinal follow-up is needed to confirm these findings. The study also found a discrepancy between self-perception of academic ability and post-UTME scores, highlighting the need for further exploration of the potential impact of self-awareness on academic and career outcomes.

Based on the findings, several recommendations are made:

Intervention programs should focus on improving emotional intelligence, especially for individuals with psychiatric morbidity. Programs strengthening family structures and enhancing parental support are recommended, as students with married parents were more likely to exhibit higher EI.

Financial stability or support should be provided to students, as increased allowances were associated with better EI outcomes.

A mixed-method study design incorporating both qualitative and quantitative approaches could provide deeper insights into the interplay between EI, family dynamics, and mental well-being. This study is one of the first to investigate the relationship between family dynamics, academic performance, and EI among fresh undergraduates in a health sciences university. However, it is cross-sectional, limiting the ability to establish causality. The self-reported nature of the data introduces the potential for bias, and concerns about identification and stigma could have influenced how participants responded to the questionnaire items.
